# Fabrication of Protein–Polysaccharide-Based Hydrogel Composites Incorporated with Magnetite Nanoparticles as Acellular Matrices

**DOI:** 10.3390/ijms26199338

**Published:** 2025-09-24

**Authors:** Anet Vadakken Gigimon, Hatim Machrafi, Claire Perfetti, Patrick Hendrick, Carlo S. Iorio

**Affiliations:** 1 Centre for Research and Engineering in Space Technologies, Université Libre de Bruxelles—ULB, Avenue Franklin D. Roosevelt 50, 1050 Brussels, Belgium; anet.vadakken.gigimon@ulb.be (A.V.G.); claire.perfetti@ulb.be (C.P.); patrick.hendrick@ulb.be (P.H.); carlo.iorio@ulb.be (C.S.I.); 2 Institute of Materials Research IMO-IMOMEC, Hasselt University, 3500 Hasselt, Belgium; 3 Aero-Thermo-Mechanics Department, Université Libre de Bruxelles—ULB, 1050 Brussels, Belgium

**Keywords:** protein–polysaccharide, hydrogel composites, biomaterials, self-healing, tissue engineering, nanoparticles, mechanical stability

## Abstract

Hydrogels with protein–polysaccharide combinations are widely used in the field of tissue engineering, as they can mimic the in vivo environments of native tissues, specifically the extracellular matrix (ECM). However, achieving stability and mechanical properties comparable to those of tissues by employing natural polymers remains a challenge due to their weak structural characteristics. In this work, we optimized the fabrication strategy of a hydrogel composite, comprising gelatin and sodium alginate (Gel-SA), by varying reaction parameters. Magnetite (Fe_3_O_4_) nanoparticles were incorporated to enhance the mechanical stability and structural integrity of the scaffold. The changes in hydrogel stiffness and viscoelastic properties due to variations in polymer mixing ratio, crosslinking time, and heating cycle, both before and after nanoparticle incorporation, were compared. FTIR spectra of crosslinked hydrogels confirmed physical interactions of Gel-SA, metal coordination bonds of alginate with Ca^2+^, and magnetite nanoparticles. Tensile and rheology tests confirmed that even at low magnetite concentration, the Gel-SA-Fe_3_O_4_ hydrogel exhibits mechanical properties comparable to soft tissues. This work has demonstrated enhanced resilience of magnetite-incorporated Gel-SA hydrogels during the heating cycle, compared to Gel-SA gel, as thermal stability is a significant concern for hydrogels containing gelatin. The interactions of thermoreversible gelatin, anionic alginate, and nanoparticles result in dynamic hydrogels, facilitating their use as viscoelastic acellular matrices.

## 1. Introduction

Tissue engineering is one of the most effective approaches for regenerating tissue defects, particularly in cases where early detection and natural regenerative capacity are limited. Unlike traditional methods that replace damaged tissue, tissue engineering aims to regenerate and repair the original tissue. Tissue defects can result from aging, chronic and acute diseases, or mechanical injuries, leading to both structural and functional alterations. During these changes, autonomous healing in biological tissues relies on a coordinated response of numerous healing constituents, including polymers, proteins, and cells. Likewise, tissue-engineering technology leverages temporary scaffolds (three-dimensional structures) that support tissue regeneration. The scaffold can be used alone or in combination with cells and growth factors to guide the healing process. The choice of scaffold material and design depends heavily on the tissue of interest. This is because tissue properties are primarily determined by the extracellular matrix (ECM). Hence, choosing scaffold materials that closely mimic the structure and function of the native ECM is essential for practical applications.

In the realm of tissue engineering, most connective and epithelial tissue matrices are considered as composite load-bearing gels [1,2]. This idea suggests that viscoelastic scaffolds like hydrogel composites could mimic the properties of these tissue matrices. A hydrogel composite material typically consists of two components: an organic component and an inorganic component. The primary component of this composite, the organic component, is very often a gel matrix. Whereas, nanomaterials, fibers, and a few polymers utilized as fillers contribute to the inorganic part of the hydrogel composite [3,4]. Gel matrices have gained attention due to their three-dimensional network structure, high water absorption capacity, and versatility. The hydrophilic polymer chains account for the high degree of water absorption, creating a moist environment similar to tissues [5]. Natural hydrogels are an optimal selection for applications where biocompatibility is crucial. They offer a range of fundamental properties, including biodegradability and non-toxicity, and are famed for their cell attachment properties. The protein–polysaccharide mixtures are of particular interest because the protein part can mimic the elastic component, and the polysaccharide constituent covers the viscous properties of the ECM [6,7]). These combinations are adequate to replicate a tissue ECM fully. For example, the ECM of connective tissues like cartilage primarily consists of water, proteins, and negatively charged biopolymers [1,8,9]. Moreover, these mixtures enhance the functionalities of the single biopolymer system by combining polymers with different characteristics.

Gelatin and alginate are among the most efficient biomimetic materials employed in developing protein-based and polysaccharide-based components, respectively [10,11]. Gelatin is derived from the partial hydrolysis of collagen, a major structural protein in cartilage. Gelatin is a thermo-responsive polymer that undergoes gel–sol transition at body temperature, during which a transition occurs from random coiled chains to right-handed triple helices. Below this temperature, helix formation and helix self-assembly are possible due to hydrogen bonds, leading to a thermo-reversible cold-set gel. Hence, this offers the self-healing advantage for minimally invasive restoration of damaged sites. The benefits of selecting gelatin as a substitute for other polymers include the presence of binding moieties crucial for cell attachments. This latter property is possible due to the ability of the gelatin to promote bioactive Arginine–Glycine–Aspartic (RGD) regions along its peptide sequence framework, mimicking the extracellular matrix [12,13]. Depending on the manufacturing conditions, the amino acid profile and thus the resulting isoelectric point (type A: isoelectric point (pI) 7–9 and type B: pI 4.8–5.5) varies [12,14]. Gelatin is widely available and relatively inexpensive, making it a cost-effective choice for large-scale applications. Gelatin is a polyampholytic polymer often combined with polysaccharides like alginates to improve its properties. Alginates are naturally occurring anionic polymers derived from brown marine algae and bacteria. Alginates are bio-inert, non-toxic, and biodegradable in the human body [15,16]. They are used as a functional material for cell encapsulation and drug carrier due to their structural similarity to extracellular matrices of living tissues [17]. Alginate possesses strong hydrophilicity due to numerous hydroxyl and carboxyl functional groups in mannuronic and guluronic acid units. The ratio of mannuronic acid (M) to guluronic acid (G) residues varies depending on the source and controls the physical properties of alginate. One of the essential properties of alginate is the ability to form an ionic gel in the presence of polyvalent cations. The ion exchange between polyvalent cations and monovalent alginate ions, followed by the coordination of metal ions, results in the gelling [18,19]. Calcium is one of the most used divalent cations for this ionic crosslinking. Calcium alginate gelation occurs in three steps, starting with mono-complex formation, followed by an egg box dimer. Then the egg box association gives multi-complexes, resulting in a gel [20]. The higher the number of G residues, the stronger the ionic crosslinking. Hence, the strength of the egg box association and the stability of the gel are highly dependent on the M/G ratio of alginate.

However, achieving the mechanical properties and structural stability required for tissue engineering, solely utilizing gelatin and alginate without overlooking biocompatibility, remains a challenge. This fact raises the question of fabricating gels by using additives or fillers to overcome this drawback. Incorporating nanomaterials into the scaffold enhances their functionality. Some commonly used nanomaterials for tissue engineering include carbon-based materials, metal and metal oxide nanoparticles, bio ceramic nanoparticles, polymeric nanoparticles, and nanofibers [4]. The addition of carbon-based nanomaterials has already proven to improve the mechanical properties of hydrogels [21,22]. Nanomaterials are particularly valued for their high surface area-to-volume ratio that promotes higher binding capacity and dispersibility in solution, which can significantly influence cellular functions such as adhesion, proliferation, and differentiation [2,3]. Magnetite (Fe_3_O_4_) nanoparticles are gaining increasing attention in tissue engineering due to their biocompatibility, ease of coating and functionalization, antimicrobial property, and magnetic stimuli response [23,24]. The trivalent (Fe^3+^) and divalent (Fe^2+^) cations in magnetite have heavy and soft metal cationic behavior, respectively. Trivalent cations bind to negatively charged ligands like carboxylate groups and divalent ions poorly crosslink neutral ligands [25,26]. Hence, incorporating Fe_3_O_4_ nanoparticles in a polymeric system can enhance colloidal stability and improve the biocompatibility of nanoparticles through surface functionalization.

The primary objective of this research is to fabricate and investigate the mechanical stability of a protein–polysaccharide blend composed of gelatin and alginate before and after magnetite nanoparticle incorporation. In comparison to existing work on magnetite nanocomposite hydrogels, this work used a significantly lower concentration of nanoparticles in combination with a gelatin–alginate hydrogel solution [27,28]. During fabrication, the key reaction parameters studied include mixing ratio and crosslinking time. The effects induced by magnetite (Fe_3_O_4_) nanoparticles at a biocompatible concentration on the structural and functional performance of the hydrogel were assessed against pure Gel-SA hydrogels. However, combining gelatin, magnetite nanoparticles, and calcium with alginate creates a complex interaction pattern that facilitates the formation of a hydrogel. Along with experimental evidence, explanations of the affinity of each component for possible non-covalent interactions are also provided. The Gel-SA-Fe_3_O_4_ hydrogel was expected to show improved mechanical stability while preserving elasticity and structural integrity, making it suitable for tissue engineering applications.

## 2. Results

All the experiments were repeated for four samples of each concentration, and the mean of the data collected was used for comparison. The data are expressed as mean ± standard deviation.

Table 1 shows the mean values of measured pH data of the pure Gel and SA solutions, the 2:1 Gel: SA preparation, respectively, and the ones containing nanoparticles.

Alginate is an anionic polysaccharide, and the deprotonation of carboxyl (COOH) to carboxylate (COO^−^) occurs at pH levels higher than 3.5. The measured pH value is higher, indicating that alginate contains a negative charge in the solutions. Furthermore, the pH variation of the SA solution after magnetite addition suggests interactions between the components. The gelatin in our study is a type A gelatin with an isoelectric point around 7–9, while magnetite has an isoelectric point around 6.8. For both components, the pH value of the Gel-SA-Fe_3_O_4_ mixture is around 5.42, which is much lower than their respective isoelectric points (IEPs). Magnetite nanoparticles exist as hydroxyl groups in solution, and the surface charge depends on the solution’s final pH. In an acidic aqueous environment, protonation of the Fe_3_O_4_ surface leads to the formation of ≡(Fe–OH_2_)^+^ moieties, due to coordination and dissociation with the solvent [29,30]. Therefore, we can suggest that gelatin and magnetite nanoparticles exhibit a positive surface charge.

### 2.1. NMR Spectrum

**Figure 1:**
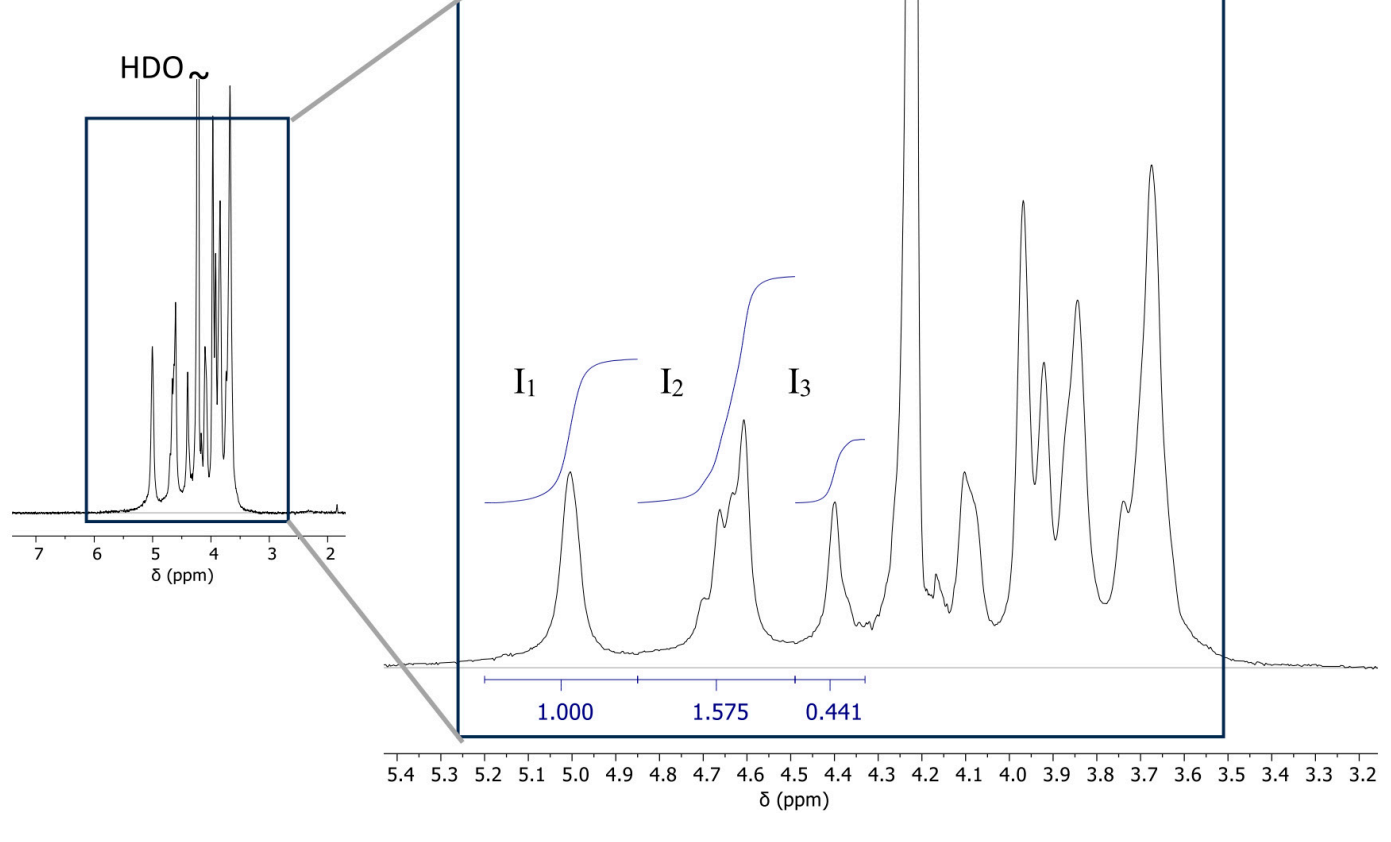
NMR spectrum of sodium alginate from Sigma Aldrich (Saint Louis, MO, USA). Intensities *I*_1_–H-1 of G, *I*_2_–H-1 of M, and H-5 of GM, and *I*_3_–H-5 of GG residues marked in the spectrum.

The ^1^H spectrum (Figure 1) agrees with the spectra of sodium alginates reported in the literature [31,32]. The M/G ratio was calculated using Equation (1). Here, *I*_1_, *I*_2_ and *I*_3_ are the intensities of H-1 of G (G-1); M-1 and GM-5 and GG-5 residues [31,33]. From the spectrum obtained, the peak values of *I*_1_, *I*_2_, and *I*_3_ are 1.00, 1.575, and 0.441. Therefore,

*F_G_* = *I*_1_/(*I*_2_ + *I*_3_) = 0.496

M/G = 1.02

The M/G ratio indicates that there are roughly equal amounts of mannuronic acid and guluronic acid residues in the chemical structure of alginate. This suggests that while M residues are available for SA-Gel polyelectrolyte and SA-Fe_3_O_4_ complex formation or stabilize iron oxide colloids, an equal amount of G residues remain available for matrices around Fe^3+^ and Fe^2+^ and selective ionic crosslinking with Ca^2+^. The gel strength depends on the interaction of alginate with multivalent cations. In high-G alginates, the high selectivity of Ca^2+^ for G-block leads to stiffer but brittle hydrogels due to the egg-box structure. The M units contribute to the formation of elastic and freeze/thaw stable gels due to flexible alginate chains [34]. According to previous studies, the flexibility of the polymer series follows the order GG < MM < MG, and in Figure 1, the NMR spectrum denotes higher intensity for MG residue, suggesting a dominant elastic behavior of the gels [35,36,37].

### 2.2. Zeta Potential

The potential for diluted magnetite dispersion and Gel-SA-Fe_3_O_4_ solution with a nanoparticle concentration of 0.02 mg/mL and pH = 7 was found to be −19.05 ± 1.18 mV and −26.08 ± 3.45 mV, respectively. The difference of −7.03 mV in potential indicates that magnetite shows improved dispersion and stability in Gel-SA-Fe_3_O_4_ mixture as a result of surface modification.

### 2.3. Tensile Strength

**Figure 2:**
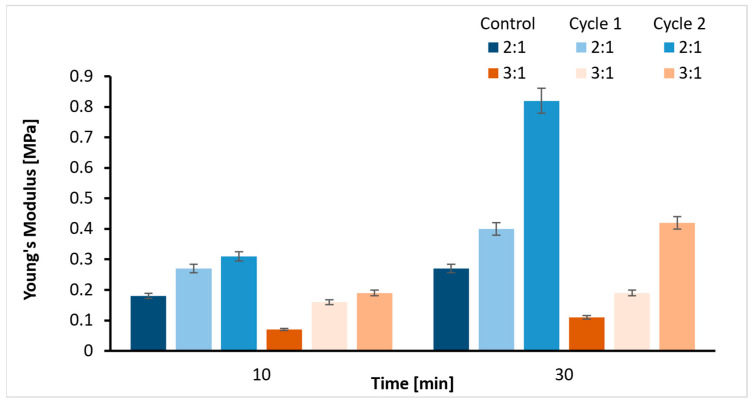
Young’s modulus values of 2:1 and 3:1 ratios with two crosslinking times (10/30 min) and heating cycles.

The Young’s modulus values obtained from the tensile test for the 2:1 and 3:1 Gel-SA hydrogel samples are plotted in Figure 2, comparing also the cases of 10 min and 30 min crosslinking times. Figure 2 illustrates an evident influence of concentration. The 2:1 Gel-SA hydrogel is stiffer with a higher *E* value than the 3:1 Gel-SA hydrogel for both crosslinking times. This is not only the case for the control sample (room temperature), but also for the samples that underwent the heating cycles. This may be due to the stable polyelectrolyte complex formation in the 2:1 Gel-SA hydrogel, resulting from electrostatic interactions between the negatively charged COO^−^ groups of alginates and the positively charged amine groups of gelatin. Regarding the crosslinking time, it appears that the hydrogel crosslinked for 30 min showed a higher *E* value. This is due to a higher degree of crosslinking, resulting in a stiffer structure.

However, it also appeared that at 10 min crosslinking time, the hydrogel showed relatively higher consistent values compared to the 30 min crosslinked gel. According to [38], salt addition screens the charges and therefore weakens the attractive electrostatic interactions between gelatin and sodium alginate molecules. Thus, a higher crosslinking time allows better crosslinking of the SA in the presence of calcium ions (Ca^2+^). Hence, the interaction with gelatin becomes weaker, and the thermal stability of the hydrogel decreases. The crosslinking time is therefore a variable to be chosen carefully. On analyzing the *E* as a function of the heating cycles, the *E* values change after each heating cycle, which suggests that heating alters the properties of the hydrogel. As the temperature increases, the polymeric chains are flexible enough for the secondary bonds to regularly break and reform, leading to continuous changes in the mechanical properties. As gelatin undergoes thermoreversible changes, a higher crosslinking time and a 3:1 hydrogel were significantly affected by temperature changes due to weaker interactions of gelatin with SA and higher gelatin content, respectively. This implies that carefully selecting the concentration and crosslinking time is insufficient to ensure both the correct *E* value and a stable structure simultaneously. One way to accommodate this is to consider the effect of adding nanoparticles.

**Figure 3:**
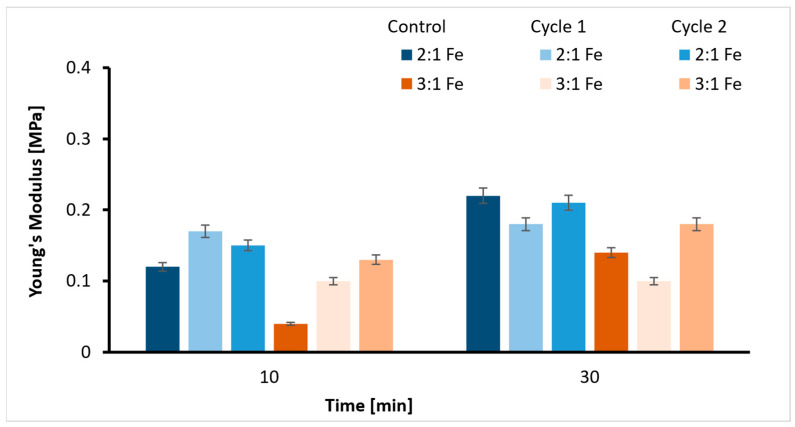
Young’s modulus values of Fe_3_O_4_ incorporated 2:1 and 3:1 ratios with two crosslinking times (10/30 min) and heating cycles.

Magnetite (Fe_3_O_4_) nanoparticles were added as additives to avoid fluctuations in the mechanical properties observed from the tensile test during heating cycles. The graph below (Figure 3) indicates that the *E* values of magnetite-added hydrogels deviate less from those of the control samples after the heating cycles compared to Figure 2, but they are relatively softer. This implies that the samples show improved thermal stability due to Fe_3_O_4_ addition, but it alters the elasticity of the material. The decrease in Young’s modulus and variations in viscoelastic properties agree with previous studies on nanocomposite hydrogels [39]. The values obtained fall in the range of Young’s modulus of soft tissues (0.1 kPa–1 MPa).

### 2.4. Rheology

**Figure 4:**
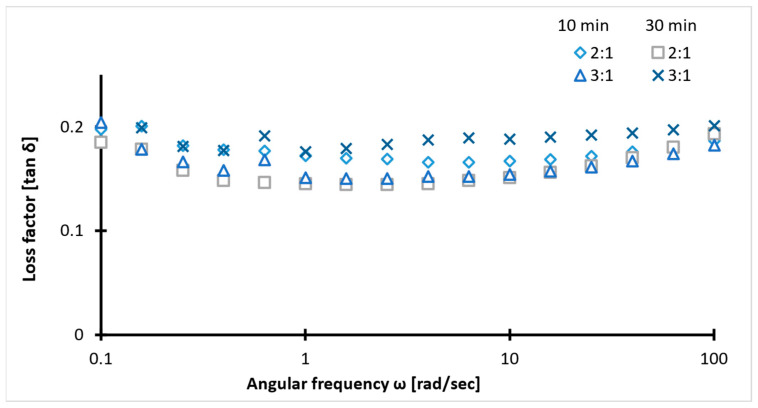
Loss factor data of 2:1 and 3:1 ratios for two crosslinking times (10/30 min).

**Figure 5:**
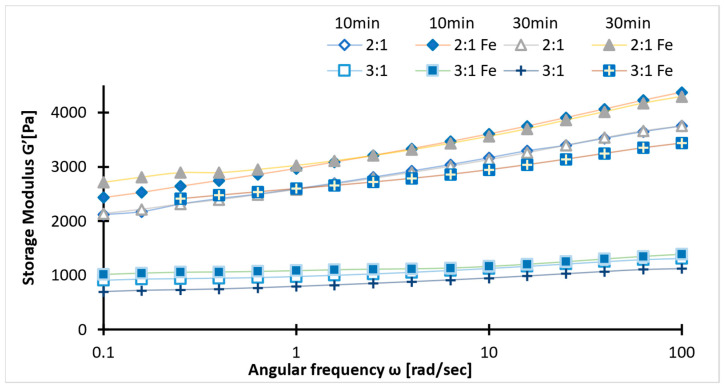
Storage modulus (G′) values of 2:1 and 3:1 ratios (with/without magnetite) for two crosslinking times (10/30 min).

**Figure 6:**
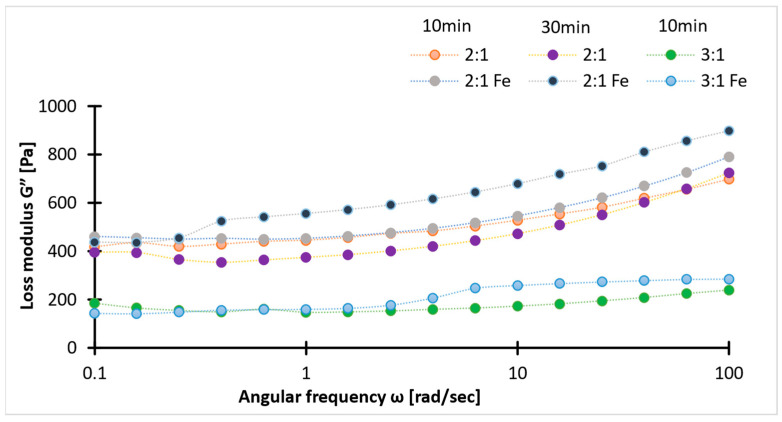
Loss modulus (G″) values of 2:1 and 3:1 ratios (with/without magnetite) for two crosslinking times (10/30 min).

**Figure 7:**
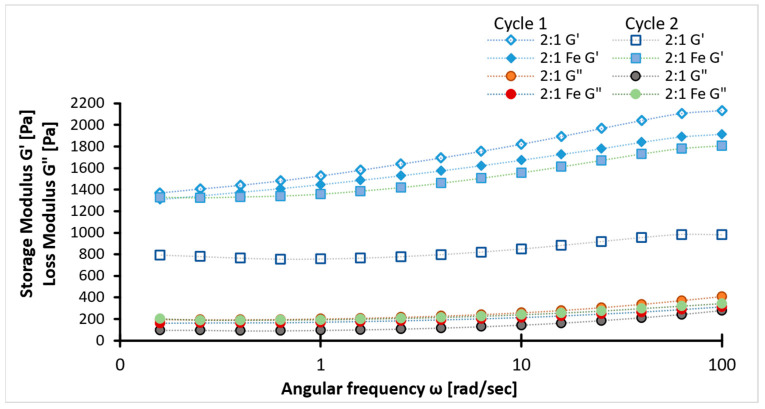
Deviations of storage (G′) and loss modulus (G″) values of 2:1 ratio (with/without magnetite) crosslinked for 10 min when subjected to heating cycles.

Viscoelastic properties of the samples were determined using a rheology test. In the figure below, tan δ across the frequency range with minor discrepancies (Figure 4), suggests stable viscoelastic behavior. The tan δ values between 0.1 and 0.2 indicate a balance between rigidity and chain flexibility. The higher values of storage modulus than loss modulus (G′ > G″) for all samples, irrespective of concentration (Figure 5 and Figure 6), confirm the superior elastic behavior of the 2:1 sample compared to the 3:1 ratio. The storage modulus (1000–4000 Pa) values of hydrogels fall within the G′ of soft tissues (10^3^–10^5^ Pa). Additionally, the Gel-SA hydrogels crosslinked for 10 min show relatively higher G′ and G″ values (Figure 5 and Figure 6) compared to the 30 min crosslinked gel for both 2:1 and 3:1 biopolymer ratios. The samples with Fe_3_O_4_ nanoparticles exhibited higher values in both storage and loss moduli compared to the hydrogels without Fe_3_O_4_, indicating an enhanced gel strength of the composite. The increase in the modulus with increasing frequency implies that these gels exhibit viscous effects and do not behave solely as solids. As expected, the storage and loss modulus values of the samples decreased after the heating cycles. This denotes that temperature changes the internal structure of the polymer. Nevertheless, the modulus values declined drastically in the hydrogel without nanoparticles. These results are in agreement with the tensile data (Figure 7). Therefore, analysis of the heating cycle data for the 10 min crosslinked 2:1 ratio confirms that nanoparticle addition resulted in a stable network structure.

### 2.5. FTIR Spectra

**Figure 8:**
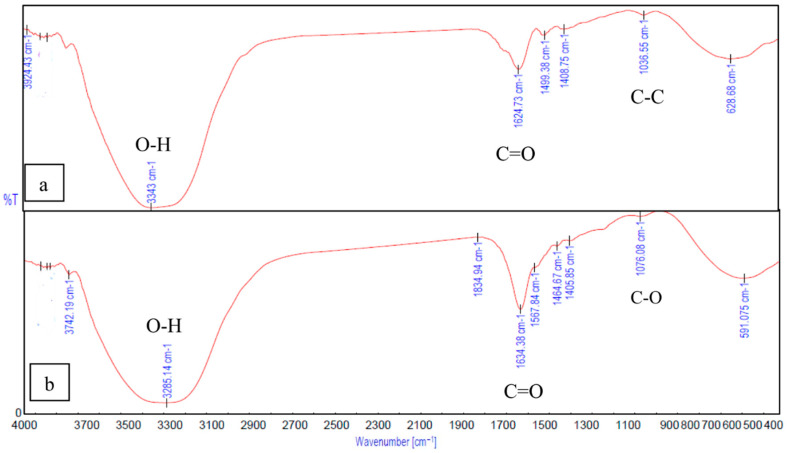
FTIR spectrum of (**a**) alginate −10 min and (**b**) gelatin.

**Figure 9:**
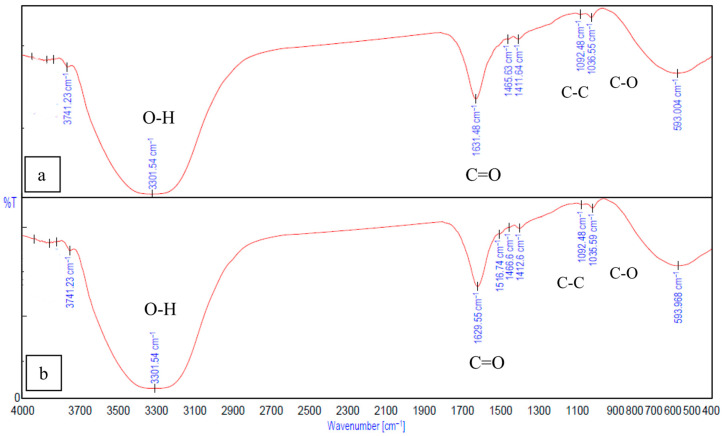
FTIR spectrum of Gel-SA, (**a**) 30 min crosslinked, (**b**) 10 min.

The interactions between gelatin and sodium alginate, the effect of crosslinking, and the binding of Fe_3_O_4_ to gelatin and alginate were confirmed and analyzed using peaks from the FTIR spectrum. In Figure 8b, the peak observed at 1634.38 cm^−1^ is from the C=O stretching vibration associated with gelatin. The peaks at 1464.67 cm^−1^ and 1567.84 cm^−1^ correspond to the functional groups of gelatin, resulting from the aliphatic C-H stretching vibration and from the combination of C=O stretching and -NH bending vibrations (amide), respectively [11,14]). In Figure 9a, the peaks at 1624.73 cm^−1^ and 1499.38 cm^−1^ correspond to the stretching vibration associated with carboxylic acid salts [14,40]) and are specific to ionic binding [41]. The peaks related to the pyranose ring of alginate can be observed within 1200–960 cm^−1^ [42]. The peaks at 3285.14 cm^−1^ and 3343 cm^−1^ (Figure 8b and Figure 8a) correspond to the O-H stretching of hydroxyl groups in gelatin and alginate, respectively.

**Figure 10:**
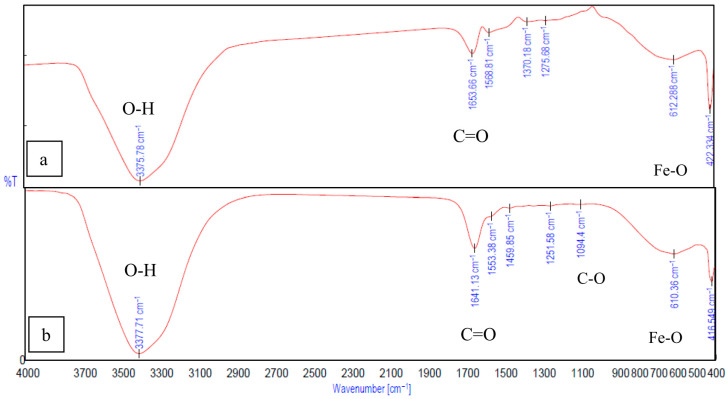
FTIR spectrum of (**a**) SA-Fe_3_O_4_ nanoparticle, (**b**) Gel-Fe_3_O_4_ nanoparticle.

**Figure 11:**
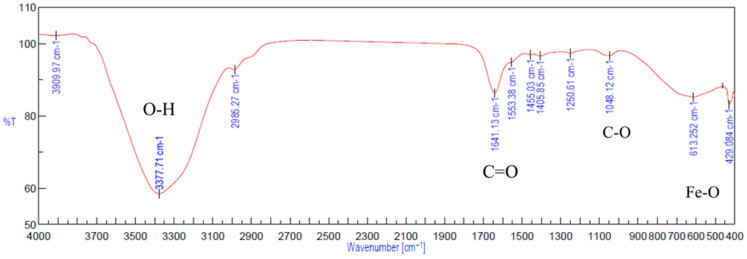
FTIR spectrum of Gel-SA-Fe_3_O_4_ hydrogel.

In the FTIR spectrum of Gel-SA hydrogel crosslinked for 10 min and 30 min (Figure 9), broadening peaks at 3301.54 cm^−1^ represent the O-H stretching band. This suggests the formation of hydrogen bonding between the polymers. The peaks at 1624.73 cm^−1^, 1499.38 cm^−1^ of alginate and the peak at 1634.38 cm^−1^ associated with amide vibration in gelatin are fused into one peak at 1629.55 cm^−1^ [41,42,43]. The peaks at 1036.55 cm^−1^ from C-C stretching observed in alginate and 1076.08 cm^−1^ from C-O stretching in gelatin are both observed, at 1035.59 cm^−1^ and 1092.48 cm^−1^ in the 2:1 Gel-SA spectrum. This confirms effective miscibility and interactions of gelatin and alginate. In the FTIR spectrum of Gel-SA crosslinked hydrogels, the peak at 1567.84 cm^−1^ of gelatin shifted to a lower wavenumber 1516.74 cm^−1^, which corresponds to -C=O stretching and -NH bending vibrations from electrostatic interactions with alginate. The complex formation of Fe_3_O_4_ with alginate is confirmed from the characteristic band of Fe-O at 422.33 cm^−1^, 612.88 cm^−1^, and the redshift of the alginate carboxyl stretching peak from 1624.73 cm^−1^ to 1653.66 cm^−1^. O-H stretching vibration observed at 3375.78 cm^−1^ confirms the hydrogen-bonded interactions. In Figure 10b, the spectrum of gelatin-Fe_3_O_4_ solution indicates the stretching of C=O and Fe-O at 1641.13 cm^−1^ and 416.549 cm^−1^ [44]. In Figure 11, the peak at 429.084 cm^−1^ confirms the presence of magnetite nanoparticles in Gel-SA-Fe_3_O_4_ hydrogel. IR peaks in the range 1200–1800 cm^−1^ and below 800 cm^−1^ suggest that magnetite particles are polymer-coated.

## 3. Discussion

### 3.1. Gel-SA Interactions

The pH test confirms that the net charge on gelatin is positive due to the NH_3_^+^ groups present in the amino acid chain, as a result of protonation. Alginate has a negative charge due to the presence of hydroxyl (-OH) and carboxyl (-COO^−^) groups. This implies that attractive electrostatic interactions and hydrogen bonds stabilize Gel-SA hydrogel (seen in FTIR data).

Due to the higher biopolymer concentration in a 2:1 ratio, the 2:1 Gel-SA hydrogel composite was expected to have a greater number of polymer–polymer interactions, including entanglements. The YM data, along with G′ and G″ values of a 2:1 Gel-SA ratio, for both crosslinking times, support this hypothesis. The 3:1 concentration has a higher gelatin content, and this can lead to a decrease in the orderliness of the resulting hydrogel due to increased charge repulsion and aggregation of gelatin molecules. Alginate forms a polyelectrolyte complex with gelatin. This limits the chances of effective ionic crosslinking with polyvalent cations, within 10 min of crosslinking. In case of longer crosslinking time, complex formation with calcium chloride screens the charges of alginate and weakens the attractive electrostatic interactions with gelatin. This could lead to gelatin leakage during the heating cycles, rendering the structure unstable due to a too low gelatin concentration and therefore of less use for medical applications. Moreover, the M/G ratio calculated from the NMR spectrum, tensile, and rheology data confirms the formation of elastic gels. Most of the previous work on the mechanical strength of alginate composites is based on M/G ratios lesser or greater than 1 and limited for M/G ratio of 1:1, as the former results in tougher gels, but in this work, we aim for highly flexible and strong gels rather than tougher ones [39,45,46]. The higher the M residues, the lower the chain stiffness of alginate hydrogels due to reduced crosslinking efficiency with Ca^2+^, but the higher the storage modulus and gel strength [47].

The concentration and crosslinking time also contribute to the changes during the heating cycle. Non-covalent interactions, such as hydrogen bonds between COOH and OH, NH_2_ and OH groups in gelatin and alginate, respectively, are one of the main mechanisms of gel development, self-healing, and structural stability. These are easily disturbed at high temperatures. The higher the amount of alginate, the higher the interactions of gelatin and the restricted mobility of gelatin chains. This explains the increased mechanical stability observed in the 2:1, 10 min crosslinked hydrogel. The low and stable tan δ values in the rheology test confirmed the dominant viscoelastic behavior of 2:1, 10 min crosslinked hydrogels.

### 3.2. Interactions of Gel-SA with Fe_3_O_4_ Nanoparticles

During solution preparation for the pH test, it was observed that nanoparticles from agglomeration or precipitation are controlled when mixed in a gelatin–alginate solution. However, the magnetite nanoparticles agglomerated at the bottom of the beaker in pure gelatin solution. The zeta potential value −26.08 ± 3.45 mV of diluted Gel-SA-Fe_3_O_4_ solution ensures the stability of magnetite in the mixture. The stability is also dependent on the concentration of polymers in the composite. A previous study indicates that natural organic ligands, when present in sufficient concentrations, may successfully compete with precipitation; however, the iron hydrolysis behavior is strongly dependent on the solution’s Ph [30]. This can be confirmed from further comparison of the pH values and FTIR spectrum of hydrogel solutions. The pH values of the SA-Fe_3_O_4_ solution (7.42) and the Gel-Fe_3_O_4_ mixture (5.26) suggest significant changes in the ionic environment of magnetite. The decrease in the C=O stretching peak intensity observed in the FTIR spectrum of SA-Fe_3_O_4_ and Gel-Fe_3_O_4_ (Figure 10) suggests that magnetite nanoparticles interact with the biopolymers, rather than simply acting as a filler in the Gel-SA-Fe_3_O_4_ mixture. In the SA-Fe_3_O_4_ solution, at a pH close to the IEP of magnetite, Fe(OH)_3_ becomes significant. Weak attractive interactions of Fe^3+^ with negatively charged alginate groups (COO^−^), release OH^−^, and slightly increase the pH (Table 1) or ion–dipole attractive interactions exist between the deprotonated hydroxyl groups of the magnetite surface (Fe-O peak Figure 10a) and the hydroxyl groups of the alginate. In the Gel-Fe_3_O_4_ solution, ≡Fe–OH_2_^+^ moieties are dominant due to protonation of the Fe_3_O_4_ surface. As both components are positively charged, this leads to electrostatic repulsion, limiting interactions and agglomeration. The hydroxyl groups on the magnetite surface can interact with -NH_2_ and -COO^−^ groups of gelatin (from pH and FTIR Figure 10b). Hence, the Gel-SA-Fe_3_O_4_ mixture enables electrostatic attractive interactions while balancing the pH and the surface charge of polymers and magnetite.

**Figure 12:**
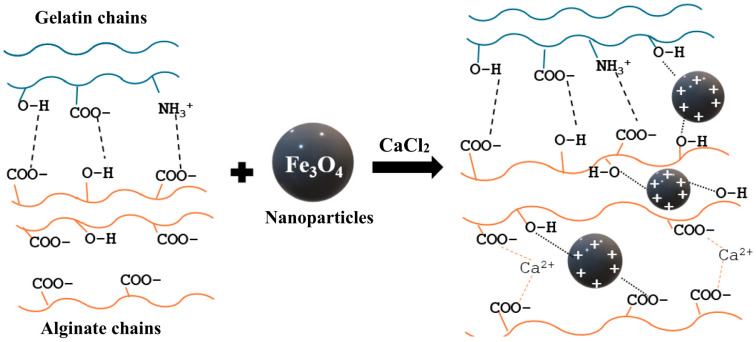
Representation of electrostatic Interactions and hydrogen bonds in Gel-SA hydrogel and coordination complexes with magnetite nanoparticle (crosslinked).

The consistency of YM values across heating cycles and the increase in storage modulus (G′) and loss modulus (G″) from rheology tests confirm that magnetite nanoparticles enhance thermal stability and strengthen the network structure. On the contrary, the YM values of the hydrogels decrease with the addition of Fe_3_O_4_. This might be due to fewer SA-Ca^2+^ and interruptions in Gel-SA interactions. Prior mixing of magnetite and Gel with SA before CaCl_2_ addition favors the interactions of alginate binding sites with magnetite and gelatin, thereby decreasing the charge density on alginate. The M/G ratio calculated also supports this. Once M and G binding sites are occupied by gelatin and magnetite, the crosslinking efficiency for Ca^2+^ is reduced. Interactions of alginate with gelatin, Fe (II, III), and Ca^2+^ in the aqueous solution control the gelation and thermal stability of the hydrogel. In comparison to the non-covalent interactions in Gel-SA hydrogel and interactions with Ca ^2+^ ions (Figure 12), the metal coordination complexes formed with Fe (II, III) in Gel-SA-Fe_3_O_4_ are stronger. The filler restricts polymer chain mobility through physical interactions and prevents premature collapse of the Gel-SA-Fe_3_O_4_ scaffold during heating. Previous works on magnetite-incorporated hydrogels have used nanoparticle concentrations of 1 wt% and above to achieve a modulus of around 0.2 MPa. Here, a concentration of one-tenth (0.1 wt%) resulted in a hydrogel with a modulus of 0.1 MPa.

## 4. Materials and Methods

### 4.1. Materials

For this work, sodium alginate (brown algae; MW: 1.2 × 10^4^ g/mol, SA) and calcium chloride dihydrate (CaCl_2_) were purchased from Sigma-Aldrich (Saint Louis, MO, USA). Gelatin (type A—porcine skin; MW: 180.1559 g/mol, Gel) was purchased from VWR Life Science AMRESCO (Solo, OH, USA). Magnetite (Fe_3_O_4_) nanoparticles (50–100 nm; MW: 231.53 g/mol) were purchased from Sigma-Aldrich (Saint Louis, MO, USA).

### 4.2. Synthesis of Gelatin–Sodium Alginate Hydrogel (Gel-SA)

To increase intermolecular interactions of ampholytic gelatin with alginate and to replicate the ECM composition, a higher concentration of gelatin was used for hydrogel synthesis. Therefore, as shown in the literature, concentration ranges of 5–10% and 1–5% were used for gelatin and alginate, respectively. To study the effect of concentration on the mechanical properties, three ratios were selected for comparison: 2:1, 3:1, and 4:1. The 2:1 ratio was synthesized by preparing a 10% (*w*/*v*) water solution of gelatin at 50 °C. To this solution, 5% (*w*/*v*) SA was added, and the mixture was stirred for 30 min at 50 °C until it became homogeneous. To prepare the 3:1 and 4:1 ratios, the same procedure was followed by varying the concentration of alginate in the solution to 3.3 and 2.5% (*w*/*v*), respectively. The 4:1 hydrogels were highly fragile due to non-homogeneous gelation and crosslinking. Therefore, they were not considered for further characterization. All the samples remained immersed in 2.5% (*w*/*v*) aqueous calcium chloride solution for either 10 min or 30 min to crosslink, to analyze the effect of these two crosslinking times. Finally, the hydrogels were washed 3 times with distilled water to remove any unreacted ions.

### 4.3. Addition of Magnetite Nanoparticle (Gel-SA-Fe_3_O_4_)

To synthesize nanoparticle-containing hydrogels, 0.1% (*w*/*v*) Fe_3_O_4_ nanoparticles were added to the Gel-SA solution before crosslinking. The solution was stirred for 30 min at 50 °C after nanoparticle addition. The 2.5% (*w*/*v*) calcium chloride solution was added to allow crosslinking for the two crosslinking times.

### 4.4. Variables in Hydrogel Formulation

As discussed, four variables were used concerning the formulation of the hydrogel: polymer concentration (Gel: SA at 2:1 and 3:1), crosslinking time (10 and 30 min), and nanoparticle addition. The concentration, crosslinking time, and addition of nanoparticles are intrinsic to the preparation process discussed earlier. The number of heating cycles (none, 1, and 2) was a fourth variable considered in this work. The purpose of this variable was to assess the mechanical stability of the hydrogel as a function of heating cycles. For the heating cycle experiment, each sample was heated in an oven to ensure uniform heating. Once the oven reached the desired temperature (40 °C ± 1 °C), the samples were heated for a fixed amount of time (10 min), and the temperature of both the oven and the samples was monitored in real-time using a thermocouple for precision. The samples were allowed to cool down (10 min) before performing the tests. To compare the mechanical properties of the hydrogels, two criteria were used: the stiffness and viscoelastic property. These were quantified using the elastic modulus (tensile), the storage and loss moduli (rheology). The mechanical properties of hydrogel composites were compared before and after the heating process. Control samples were used for each experiment for this comparison. Cycle 1 denotes heating the sample once, and cycle 2 indicates heating the sample a second time. Table 2 presents a summary of the variables considered in this work.

### 4.5. Characterization

#### 4.5.1. NMR Spectroscopy

The Nuclear Magnetic Resonance (NMR) spectroscopy was carried out to determine the mannuronic acid to guluronic acid (M/G) residue ratio. This ratio influences the alginate block structure and significantly impacts the gel strength, thereby affecting the mechanical properties of the hydrogel. For the NMR sample preparation, 5 mg of sodium alginate was dissolved in 700 µL D_2_O under stirring at 50 °C. The solution was then transferred to a standard 5 mm NMR tube. The ^1^H NMR spectrum was recorded at 80 °C on a JEOL JNM-ECZ600R/S3 spectrometer (JEOL RESONANCE, Tokyo, Japan) operating at 14.1 Tesla (600.17 MHz for ^1^H) equipped with a 5 mm NM Royal probe^TM^ and using the following acquisition parameters: 20 ppm spectral window centered at about 4.5 ppm, 8.7 s relaxation delay, 2.6 µs (30°) RF pulse, 1.1 s acquisition time, four dummy scans and 32 scans. Processing with MestReNova software (14.3.1-31739) included apodization of the free induction decay (sine-squared II -50% 50%), zero filling (up to 256k points), Fourier transform, phase, and baseline corrections. The HDO signal was used for chemical shift referencing (4.22 ppm at 80 °C). Integrals were measured using automatic linear corrections. The M/G ratio was calculated from Equations (1) and (2).

(1) $$\frac{M}{G}=\frac{\left(1-F_{G}\right)}{F_{G}}$$

(2) $$F_{G}=\frac{I_{1}}{\left(I_{2}+I_{3}\right)}$$

where $F_{G}$ is the molar fraction of G residue, $I_{1}$, $I_{2}$, and $I_{3}$ denote the intensities of the lines in the spectrum.

#### 4.5.2. Zeta Potentiometer

The zeta potential measurements were performed using the Zetasizer Nano ZS from Malvern Instruments (Malvern Panalytical, Malvern, United Kingdom). The measurements were performed for magnetite nanoparticle aqueous dispersion and Gel-SA-Fe_3_O_4_ solution with a concentration of at 0.2 mg/mL 25 °C in a folded capillary cell. The equipment, with the aid of Malvern Zetasizer software v8.01, determines the electrophoretic mobility of charged particles by measuring the particle velocity under an applied electric field. Then, from the obtained electrophoretic mobility, the zeta potential of the particles is calculated. The average of six potential values obtained from each solution was used for comparison.

#### 4.5.3. Tensile Test

**Figure 13:**
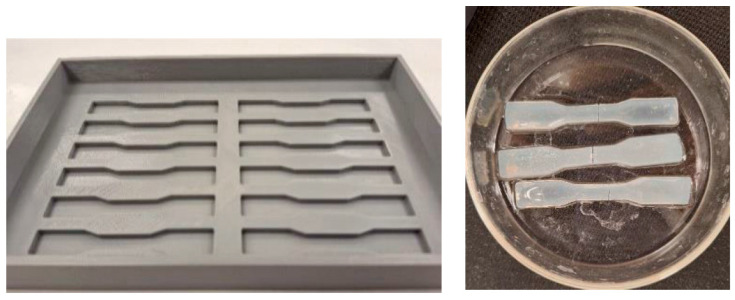
Dog-bone-shaped mold and sample for tensile test.

The mechanical strength of the synthesized hydrogels was tested using the Uniaxial SHIMADZU AUTOGRAPH AGS-X (Columbia, MD, USA) tensile machine with a crosshead speed of 0.02 mm/s. The tensile tests were carried out at room temperature on dog-bone-shaped samples made from a Teflon mold (Figure 13), as per the ASTM D412 standard for dumbbell and straight section specimens, used on a tabletop universal testing machine [48].

The tensile machine measures load (*F*), displacement (∆l) and the stress (σ) and strain (ε) values were calculated with the following Equations (3) and (4):

(3) σ=FA

(4) $$\varepsilon =\frac{∆l}{l_{0}}\times 100$$

where *σ* refers to the stress, *A* is the sample inner area (perpendicular to the direction of force) calculated as “thickness × width”, *ε* refers to the strain and the initial (*l*_0_) length of the sample. Young’s modulus (*E*) or modulus of elasticity was calculated from the slope of the linear part of the stress–strain curve.

(5) E=σε

The Young’s modulus was calculated for the control samples as well as the heated samples, after each heating process.

#### 4.5.4. Rheometry

The viscoelastic properties of the hydrogel were tested using an Anton Paar MCR 302e rheometer (Graz, Austria). To measure the viscoelasticity of the synthesized hydrogels, frequency (0.1–100 rad/s) dependent oscillatory tests were employed. The parallel plates (PP) model with a diameter of 25 mm was used for conducting frequency sweep tests. The storage modulus G′ represents the elastic portion of the viscoelastic behavior, which quasi describes the solid-state behavior of the sample. The loss modulus G″ characterizes the viscous portion, which can be seen as the liquid-state behavior of the sample. These moduli are given by:

(6) $$G^{\prime }\;=\frac{\sigma }{\varepsilon }\;Cos\;\delta$$

(7) $$G^{\prime \prime }=\frac{\sigma }{\varepsilon }\;Sin\;\delta$$

The loss angle (*δ*) represents the deviation from viscoelastic behavior. It is the ratio of loss modulus to storage modulus, given by:

(8) tanδ= G″G′

The 2:1 and 3:1 concentrations of Gel-SA and Gel-SA-Fe_3_O_4_ hydrogels were used for the rheology tests to study the viscoelastic properties of hydrogels. From this, the heating cycle data of the hydrogel ratio with better mechanical performance will be tested and analyzed.

#### 4.5.5. FTIR Spectroscopy

The molecular structure of the hydrogels was analyzed using Fourier transform infrared (FT-IR) spectroscopy. The FTIR of hydrogels with and without magnetite was analyzed to identify interactions between gelatin, sodium alginate, and magnetite nanoparticles. The bond peaks were examined using an FTIR Jasco 6600 (Tokyo, Japan) with a wavelength range from 400 to 4000 cm^−1^. For the samples without Fe_3_O_4_, attenuated total reflectance (ATR) spectroscopy measurements were performed using a diamond crystal. However, Germanium crystal was used for the samples with Fe_3_O_4_, as they were darker and more opaque.

## 5. Conclusions

In this work, a magnetite nanoparticle-incorporated protein–polysaccharide combination is fabricated via a simple synthesis procedure, where the mixing ratio of biopolymers, crosslinking time, and heating cycle are varied. In a 2:1 ratio, the higher concentration of alginate reduces the mobility of gelatin chains due to increased electrostatic interactions, and the affinity of alginate to the cross-linker (CaCl_2_) makes the gelling of the hydrogel highly stable even during heating cycles. Consequently, 10 min crosslinked hydrogels show higher stability than samples crosslinked for longer durations. Therefore, a 10 min crosslinked 2:1 Gel-SA hydrogel exhibits higher structural stability at temperatures above 37 °C. In comparison to existing work on magnetite nanocomposite hydrogels, this work achieves comparable mechanical properties with a significantly lower concentration of nanoparticles in combination with a gelatin–alginate hydrogel solution. Incorporating Fe_3_O_4_ nanoparticles into a Gel-SA hydrogel yields multifunctional hydrogel composites with a stable network structure and enhanced temperature tolerance. This hydrogel is specifically beneficial for ECM applications in soft tissues such as muscles or skin, as the stiffness and viscoelasticity achieved match the mechanical properties of these tissues.

## Figures and Tables

**Table 1:** pH data of hydrogel solutions.

**Hydrogel Composition**	**Mean pH Values (SD = ±0.12)**
Pure gelatin solution (10% *w*/*v*)	5.33
Pure SA solution (5% *w*/*v*)	7.24
2:1 Gel:SA	5.55
**Addition of Fe_3_O_4_ Nanoparticle**	**Mean pH Values (SD = ±0.12)**
Gel + Fe_3_O_4_ 0.1% (*w*/*v*)	5.26
SA + Fe_3_O_4_ 0.1% (*w*/*v*)	7.42
2:1 Gel:SA + Fe_3_O_4_ 0.1% (*w*/*v*)	5.42

**Table 2:** The variables considered in the hydrogel fabrication: heating cycle, hydrogel components, weight ratio, and crosslinking time.

Heating 40 ± 1 °C	Hydrogel		Weight Ratio		Crosslinking Time	
No heat/Cycle 1/Cycle 2	Gel: SA	Gel: SA: Fe_3_O_4_	2:1	3:1	10 min	30 min

## Data Availability

Data are available from the authors upon reasonable request.
